# Identification of mutation resistance coldspots for targeting the SARS‐CoV2 main protease

**DOI:** 10.1002/iub.2465

**Published:** 2021-03-22

**Authors:** Navaneethakrishnan Krishnamoorthy, Khalid Fakhro

**Affiliations:** ^1^ Department of Human Genetics Sidra Medicine Doha Qatar; ^2^ Department of Genetic Medicine Weill Cornell Medical College Doha Qatar; ^3^ College of Health and Life Sciences Hamad Bin Khalifa University Doha Qatar

**Keywords:** dimer interface, mutation hotspot, mutation‐based drug resistance, structure–function relationship, surface coldspots, X‐ray structure

## Abstract

Mutations in the novel coronavirus SARS‐CoV2 are the major concern as they might lead to drug/vaccine resistance. In the host cell, the virus largely depends on the main protease (M^pro^) to regulate infection hence it is one of the most attractive targets for inhibitor design. However, >19,000 mutations in the M^pro^ have already been reported. The mutations encompassing 282 amino acid positions and these “hotspots” might change the M^pro^ structure, activity and potentially delay therapeutic strategies targeting M^pro^. Thus, here we identified 24 mutational “coldspots” where mutations have not been observed. We compared the structure–function relationship of these coldspots with several SARS‐CoV2 M^pro^ X‐ray crystal structures. We found that three coldspot residues (Leu141, Phe185, and Gln192) help to form the active site, while seven (Gly2, Arg4, Tyr126, Lys137, Leu141, Leu286, and Leu287) contribute to dimer formation that is required for M^pro^ activity. The surface of the dimer interface is more resistant to mutations compared to the active site. Interestingly, most of the coldspots are found in three clusters and forms conserved patterns when compared with other coronaviruses. Importantly, several conserved coldspots are available on the surface of the active site and at the dimer interface for targeting. The identification and short list of these coldspots offers a new perspective to target the SARS‐CoV2 M^pro^ while avoiding mutation‐based drug resistance.

## 1 INTRODUCTION

In SARS‐CoV2, main protease (M^pro^) or 3CL‐protease (3CL^pro^) is essential for proteolytic activity, production of structural proteins and host cell infection.^1^ We already have access to high resolution 3D‐structures of the SARS‐CoV2 M^pro^, which were developed with potential inhibitors as co‐crystals using X‐ray crystallography.^2^, ^3^, ^4^, ^5^, ^6^, ^7^ Based on these structures, we know that domains I (8–101) and II (102–185) play major roles in the formation of the active site and provide binding sites for inhibitors; while domain III (202–306) is important in the regulation of protease activity. The catalytic dyad His41 and Cys145 are located at the active site that forms in a cleft between domains I and II. Most efforts to design anti‐viral inhibitors using drug repurposing approaches are focused on targeting this active site.^1^, ^2^, ^8^ Others are working on inhibitors to target the allosteric sites^3^, ^5^ at the SARS‐CoV2 M^pro^ dimer interface that disrupts protease activity in the close relative severe acute respiratory syndrome coronavirus (SARS‐CoV).^9^ Despite these advances, various challenges such as mutation, structural plasticity and mutation‐based stability complicate drug targeting of this protease.^10^, ^11^ Mutation is a common phenomenon in viral systems and delays the identification of a vaccine/drug candidate. Early in the coronavirus disease 2019 (COVID‐19) pandemic, mutational hotspots were reported within SARS‐CoV2 genomic sequences.^12^ Modeling studies have helped to explain the dynamic molecular characteristics of mutations in SARS‐CoV2 M^pro^.^10^, ^13^ However, mutational coldspots (with no known mutations) at the molecular 3D‐level and their potential structural roles have not been examined in SARS‐CoV2 M^pro^. We believe that identifying SARS‐CoV2 M^pro^ coldspots may lead to the location of mutation‐resistance binding site(s) that are suitable targets for antiviral agents. With this in mind, we aimed to identify and understand the importance of mutational coldspots in SARS‐CoV2 M^pro^ that have shown no reported mutations at the time of collection.

## 2 MUTATIONAL HOTSPOTS AND COLDSPOTS

**FIGURE 1:**
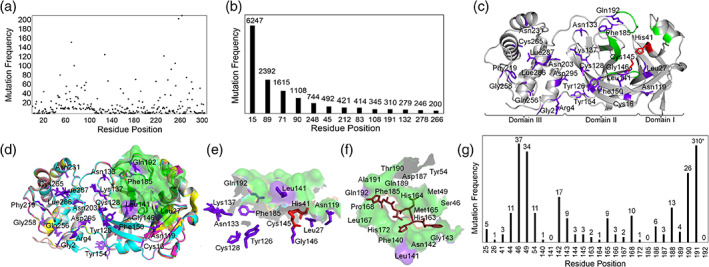
Hotspots and coldspots in SARS‐Cov2 M^pro^. (a) Residues with less than 200 mutations and (b) residues with more than 200 mutations plotted against mutation frequency. (c) Structural mapping of coldspots (PDB code: 6LU7); (d) superimposition of high‐resolution structures of SARS‐CoV2 M^pro^: PDB codes are 6LU7 (grey), 6Y2F (cyan), 6LZE (magenta), 6M0K (yellow), 7BUY (salmon); (e) coldspots in and around the active sites of the superimposed structures of SARS‐CoV2 M^pro^; (f) active site pocket in 6LU7 with inhibitor N3 (ruby). (g) Mutational frequency of active site residues. Coldspots: purple sticks; active sites: green surface/cartoon; catalytic dyads: red sticks

To identify the coldspots in SARS‐CoV2 M^pro^, we aggregated the circulating missense mutations reported in Global Initiative on Sharing All Influenza Data (GISAID) until November 2, 2020 by searching the database against the reference protein sequence Wuhan‐Hu‐1 (NC_045512.2, 10,055‐10,977) with 306 amino acid positions. This was approximately 11 months since the start of the COVID‐19 outbreak, which should have provided enough time for the virus to accumulate some key mutations for survival.^12^ The dataset contained 19,154 mutations covering total of 282 out of 306 residue positions of SARS‐CoV2 M^pro^, which are referred here as mutational hotspots (Figure 1a,b). These hotspots showed a minimum of one mutation (Figure 1a). In particular, the data (top 13 with >200 mutations, Figure 1b) showed the following hotspot positions were the most frequently mutated: Gly15 (6,297 reported mutations), Leu89 (2,392), Gly71 (1,615), Lys90 (1,108), and Asp248 (744) (Figure 1b). The remaining 24 positions had no reported mutations and were considered mutational coldspots (Figure 1c), as they have shown a degree of mutation resistance up to this stage of the pandemic. Therefore, we further studied structures of SARS‐CoV2 M^pro^ to understand the structure‐functional relevance of coldspots.

## 3 COLDSPOTS AT THE ACTIVE/INHIBITOR SITE

Next, to analyze the coldspots in and around the active site, we selected five Xray‐crystallographic structures with high resolution (Protein Data Bank [PDB] codes: 6LU7, 6Y2F, 6LZE, 6M0K, and 7BUY), that had been co‐crystalized with antiviral drug candidates.^2^, ^4^, ^7^, ^14^ However, the inhibitors were not optimal for SARS‐CoV2.^15^ We believe the non‐mutational residues (coldspots) could be appropriate target regions for designing effective inhibitors of SARS‐CoV2 M^pro^. We found 15 coldspots to be from domains I and II, and the remaining nine were in domain III (Figure 1c). The inhibitor‐binding sites in the five SARS‐CoV2 M^pro^ structures were superimposed (Figure 1d,e,f), which show that a total of 25 residues (Figure 1g) form the binding sites for the reported inhibitors (6LU7‐N3 [M^pro^‐inhibitor name], 6Y2F‐13b, 6LZE‐11a, 6M0K‐11b, and 7BUY‐carmofur). In these 25 positions, 22 were affected by a total of 525 mutations. In particular, residue positions 46, 49, 142, 190, and 191 showed more than 15 mutations each. This suggests that most of the active site residues are mutated and challenging to target.

Interestingly, we mapped three coldspots, Leu141, Phe185, and Gln192, in the 6LU7‐N3 complex (Figure 1f). The structural importance of these coldspots was emphasized by the recent X‐ray crystallographic studies of SARS‐CoV2 M^pro^ ^3^, ^4^, ^5^ demonstrating the involvement of the coldspots in the formation of substrate‐binding sites and Phe185 and Gln192 in the stability of the active site. We found coldspots Asn133 and Lys137 beneath the surface formed by the binding‐site residues (Figure 1e), specifically, Leu27, Asn119, and Gly146 are near the catalytic dyad (His41 and Cys145). They may provide some support to the catalytic center, as evidenced by a recent study, in which Leu27 was found to play a key role in the activity of the M^pro^ structure of SARS‐CoV2.^6^ Whereas, Leu27 and Asn119 are involved in the formation of the binding site in SARS‐CoV M^pro^ (Table S1). However, based on our data analysis, the other pocket‐forming residues in the structures undergo mutations, which may modify the shape of the binding pocket. This prediction is supported by a recent study,^10^ in which the structures of the mutants Met49Ile, Pro184Leu/Ser, and Ala191Val induced conformational changes. This indicates that coldspots are required at the active site to maintain effective targeting.

Importantly, in SARS‐CoV2 M^pro^, the key active site residues His41 (3 mutations), Phe140 (1 mutation), Cys145 (3 mutations), Glu166 (3 mutations), and His172 (1 mutation) showed low mutation frequencies (a total of 11 out of 525 mutations at the active site) (Figure 1g). This suggests that the residues involved in critical functions at the active site are mutated less frequently than other residues, which indicates functional importance of coldspots.

## 4 COLDSPOTS AT THE DIMER INTERFACE

**FIGURE 2:**
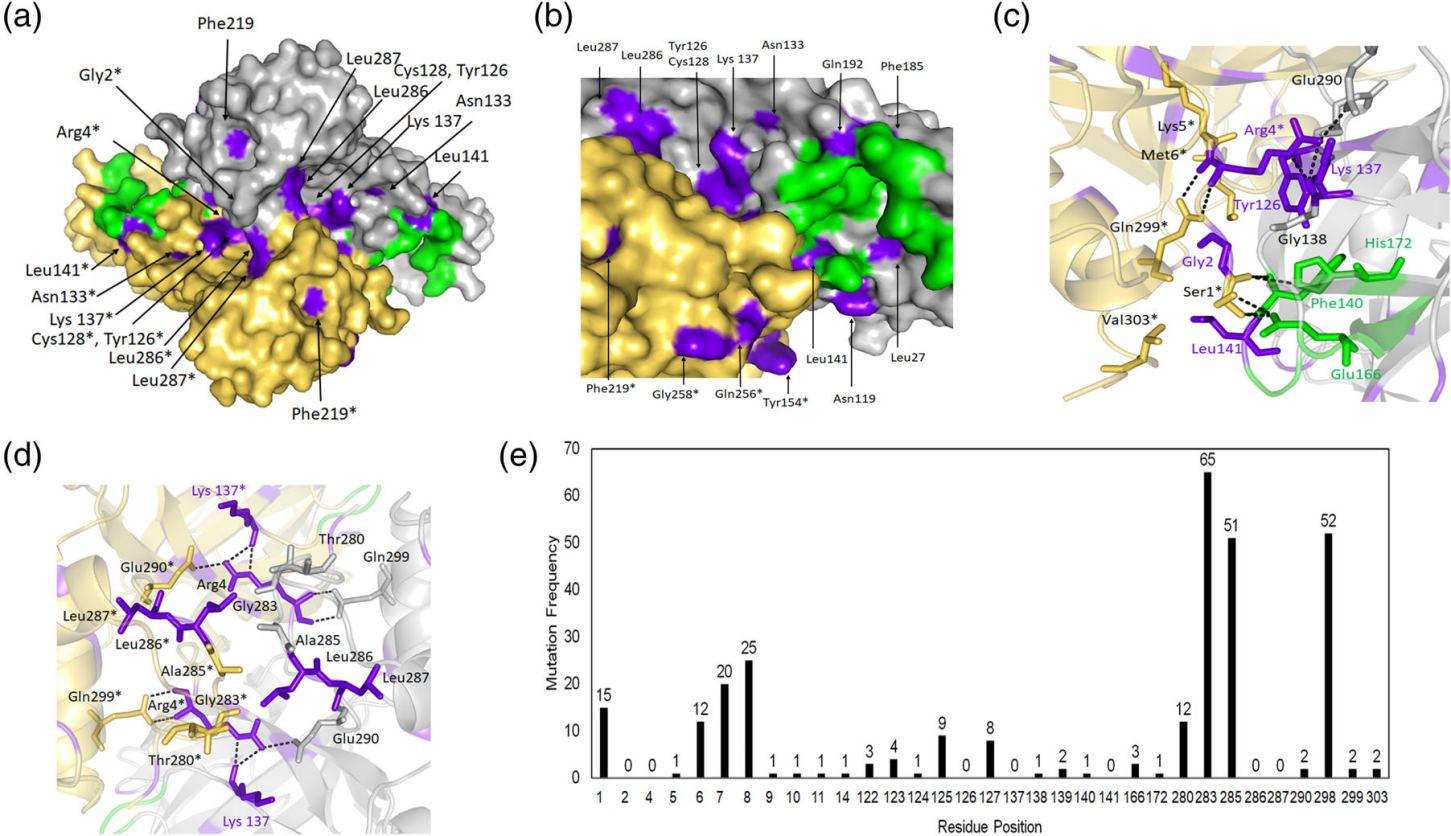
Dimer interface of SARS‐CoV2 M^pro^. (a) Surface model of the dimer (PDB code: 6LU7); (b) extended and detailed view of panel a showing the dimer interface, with coldspot residues on the surface model; (c) site1 and (d) site2 with coldspot residues and interactions involved in dimerization; (e) mutation frequency of dimer interface residues. Grey cartoon/surface: protomer A; yellow surface/cartoon: protomer B; residues with * are from protomer B; purple: coldspot residues; green: active site; dashes: hydrogen bonds

An alternate therapeutic strategy is to design antiviral agents to target the dimerization of the SARS‐CoV M^pro^, as the dimeric form is essential for activity^9^ and, with 98% identity, is also applicable to SARS‐CoV2 M^pro^.^3^, ^5^ Here, we examined the functional relevance of coldspots on the surface of the dimer interface in SARS‐CoV2 (PDB code: 6LU7) (Figure 2a). Half of the coldspot positions are on the surface of the protease (Figure 2a,b), and the rest are buried. We discovered seven coldspot positions (Gly2, Arg4, Tyr126, Lys137, Leu141, Leu286, and Leu287) on the surface that are involved in the formation of the dimer interface in the SARS‐CoV2 M^pro^ (Figure 2c,d). They form two sites: the first is based on the positions Gly2, Arg4, Tyr126, Lys137, and Leu141 (Figure 2c), and the second site includes the positions Arg4, Lys137, Leu286, and Leu287 (Figure 2d). In the SARS‐CoV M^pro^, these sites include several key interactions, Arg4‐Lys137‐Glu290, Gly2‐Arg4‐Tyr126, Ser284‐Tyr285‐L286, and Ser1‐Glu166‐His163‐His172, that have been experimentally proven to be vital for maintaining the dimer interface and the active site (Table S1).

In SARS‐CoV2 M^pro^, we observed a hydrogen bond between Arg4 and Lys137 (Figure 2c). As both are coldspots (with three other coldspots nearby, Gly2, Tyr126, and Leu141), this appears to be a potential site for inhibition. It also appears slightly similar to the one recently proposed as a potential allosteric site in SARS‐CoV2 M^pro^ ^3^ using crystal‐electrophilic screening. Residue 141 plays a dual role by forming the active site and dimer interface with Val303 (Figures 1e,f and 2c). Leu286 forms two hydrogen bonds with Ser284 in protomer A and hydrophobic interactions with Tyr280, Gly283, and Ala285 in protomer B (Figure 2d). Because they form a channel to the catalytic center and regulate the catalytic machinery in SARS‐CoV,^16^ this site could be another potential allosteric site to target. Moreover, seven other coldspot residues (Asn119, Asn133, Tyr154, Phe185, Gln192, Gln256, and Gly258) on the protease surface (Figure 2a,b) and do not contribute to the dimer interface. The other structures of SARS‐CoV2 M^pro^ also confirms the functional relevance of the coldspot residues Gly2, Arg4, Tyr126, Lys137, Leu141, and Leu286 that are directly involved in dimer formation through various interactions^5^, ^7^ (Table S1). These correlate with our hypothesis that the observed coldspots may serve as mutation‐resistant allosteric sites.

There are 21 hotspots at the interface covering 296 mutations; out of 21, only 10 hotspots had more than eight mutations (Figure 2e). The frequency of mutations was high at residue positions Gly283, Ala285, and Arg298 (65, 51, and 52 mutations, respectively), compared with the hotspots at the N‐finger (residues 1–8) region. The dimer interface mutated (296) relatively less frequently than the active site (525 mutations) (Figure 1g). This indicates that the dimer interface at SARS‐CoV M^pro^ seems to be more resistant to mutations.

## 5 COLDSPOTS CONSERVED AMONG CORONAVIRUSES

**FIGURE 3:**
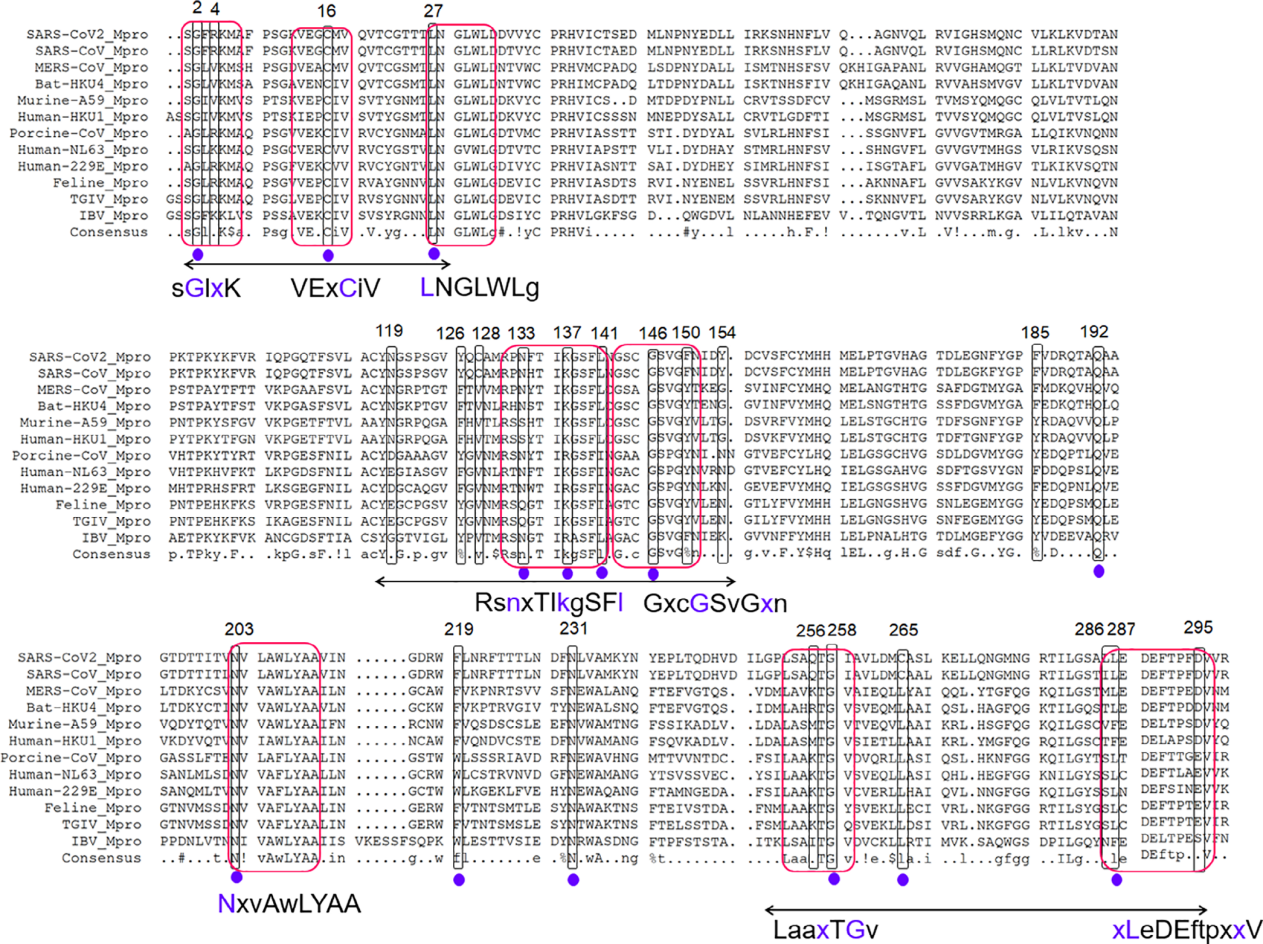
Multiple sequence alignment of M^pro^ from various coronaviruses. The mutational coldspots of SARS‐CoV2 M^pro^ are shown in boxed regions and red boxed regions are conserved patterns. The purple dots indicate conserved coldspots among all the aligned coronavirus Mpros, and double arrows indicate the coldspot clusters. Here, SARS‐CoV: severe acute respiratory syndrome coronavirus; HKU4: Tylonycteris bat coronavirus HKU4; PEDV: porcine epidemic diarrhea virus; Human‐229E: human coronavirus 229E; MERS: middle east respiratory syndrome‐related coronavirus; Murine‐A59: murine hepatitis virus strain A59; HKU1: human coronavirus HKU1 (isolate N1); Human‐NL63: human coronavirus NL63; FIPV: feline infectious peritonitis virus (strain 79–1146); TGEV: transmissible gastroenteritis virus; IBV, infectious bronchitis virus, were used in a multiple sequence alignment against M^pro^ of SARS‐CoV2

Next, we used Multalign for structure‐based sequence alignment of 12 different coronaviruses (CoVs) to represent the coronavirus superfamily (PDB codes: 2HOB, 4YOI, 4ZUH, 2ZU2, 4WME, 6JIJ, 3D23, 6FV2, 4ZRO, 2AMP, 2Q6F, 6LU7) and analyzed their degree of conservation. It shows that the majority of the coldspots are arranged as three clusters: four coldspots at the N‐terminal, six near the C‐terminal and, surprisingly, nine near the active site in domain II (Figure 3). We found 16 coldspots are distributed in eight conserved blocks including a block GxcGSvGxn based on motif GSCGS that is essential for the initiation of the catalysis in middle east respiratory syndrome‐related coronavirus (MERS‐CoV) and SARS‐CoV.^15^ Similarly, the other conserved blocks might have some functional role as they are found in the key structural regions. Interestingly, 14 out of the 24 coldspots were conserved among all the CoVs. Moreover, most of the 14 conserved coldspots of SARS‐CoV2 M^pro^ have critical roles in the formation of the active site (Leu27 and Gln192) and at the dimer interface (Gly2, Cys16, Lys137, Leu287, and Asp295), and Leu141 has both roles (Table S1, Figures 2 and 3). The significance of the other conserved coldspots (Asn133, Gly146, Asn203, Phe219, Asn231, and Gly258) in SARS‐CoV2 M^pro^ are unclear. Overall, this sequence alignment suggests that not all the highly conserved residues in SARS‐CoV2 M^pro^ are resistant to mutation. Although only certain coldspots are conserved among CoVs, most of the conserved sites contribute to the formation of critical interactions (Table S1).

## 6 BIOLOGICAL RELEVANCE

It is understood that the SARS‐CoV2 M^pro^ is undergoing or accumulating mutations, thus it is essential to identify consistent mutational coldspots that can be targeted with antiviral drugs. In addition, the data of nearly 20,000 global mutations used in this study were collected at the end of the first wave of COVID‐19, are minimal. However, the identified mutational coldspots have biological relevance, according to the high‐resolution X‐ray crystal structures of SARS‐CoV2,^3^, ^5^, ^7^, ^14^ sequence conservation among CoVs, and experimental evidence provided by the published X‐ray crystal structures of other CoV main proteases (Table S1).

The observed mutational frequencies in the hotspots at the active site and dimer interface indicate that the virus may develop protective strategies against inhibitors. This correlates with the findings described in recent reports, which show the hotspots/positions are changing the shape of the sites via mutations and plasticity.^10^, ^11^, ^17^ However, we pinpointed and proposed several conserved coldspots at the surface of the active site and dimer interface that could be optimum targets for the design of mutation‐resistance antivirals. This is evident from the fact that mutations have not been observed in the coldspots since the virus was first detected. However, further research is warranted for a deeper understanding of the phenomenon.

## CONFLICT OF INTEREST

The authors declare no competing interests.

## DATA AVAILABILITY STATEMENT

The X‐ray crystal structures that support the findings of this study are publically available in protein data bank at https://www.rcsb.org.

## Supporting information


**Table S1** Structural and functional importance of mutational coldspots in SARS‐CoV2
