# Supplementary material for: Identification of mutation resistance coldspots for targeting the SARS‐CoV2 main protease
Source: IUBMB Life. 2021 Mar 22;73(4):670–5. doi: 10.1002/iub.2465 (PMC8250577; doi:10.1002/iub.2465)
Supplement: Supplementary file 1 — Table S1 Structural and functional importance of mutational coldspots in SARS‐CoV2 [file IUB-73-670-s001.docx]

**Supplement table.**

**Table S1**: Structural and functional importance of mutational coldspots in SARS-CoV2

| S.no | Coldspot residue position | Structural and/or functional role | Publication | Experimental method |
| --- | --- | --- | --- | --- |
| 1 | 2, 4, 137  141 | Gly2, Arg4 and Lys137 in the binding pocket at the dimer interface of SARS-CoV2 M^pro^ for the inhibitor POB0073.  141 is involved in the formation of the binding site at the dimer interface of SARS-CoV2 M^pro^ for the fragment/inhibitor Z264347221 | Douangamath et al.,(1) | Crystallographic and electrophilic fragment screening |
| 2 | 16 | Cys16 Forms hydrogen bonds with Ser10, Gly11, and Glu14 in SARS-CoV2 M^pro^ at the dimer interface | Kneller et al.,(2) | Neutron X-ray crystallography |
| 3 | 27 | Leu27 is involved in the activity of SARS-CoV2 M^pro^. It interacts with active binding probe, Biotin-PEG(4)-Abu-Tle-Leu-Gln-VS (B-QS1-VS) | Rut et al.,(3) | Synthesis and X-ray crystallography |
| 4 | 192, 185, 141, 2, 4, 126, 137, 141, 286 | Leu141, Phe185, Gln192 are involved in formation of inhibitor N3-binding pocket, and Gly2, Arg4, Tyr126, Lys137, Leu141, Leu286, and Leu287 function in the dimerization of SARS-CoV2 M^pro^ | Jin et al., (4) | X-ray crystallography |
| 5 | 286 | In SARS-CoV2 M^pro^, Ala285 and Leu286 induced slightly closer packing at the dimer interface and increased catalytic activity (compared to SARS-CoV M^pro^) | Zhang et al.,(5) | X-ray crystallography |
| 6 | 192 | In MERS-CoV infected mice, residue 192 in M^pro^ interacts with compound 6h and has a potential specificity role in selection of inhibitors 7j and 7i | Rathnayake et al.,(6) | Therapeutic treatment in a mouse model, X-ray crystallography |
| 7 | 185, 192 | In SARS-CoV2 M^pro^, a long loop Phe185-194 is stabilized by the interactions of 189-192-186. Phe185 and Leu167 form a deep hydrophobic pocket for substrate binding | Kneller et al.,(2) | Neutron X-ray crystallography |
| 8 | 2, 4, 16, 119, 137, 141 | In SARS-CoV2 M^pro^ at the dimer interface, N-terminal residues 1-16 (N-finger) interact with 118 (in protomer B)-125 (B) and loop 137 (B) -142 (B) | Kneller et al.,(2) | Neutron X-ray crystallography |
| 9 | 4, 27, 141, 185, 295 | Glu185 (in PEDV M^pro^) and Val185 (in SARS-CoV M^pro^) located in motif 3 of the substrate binding pocket. In PEDV M^pro^, Leu27 and Asn141 are two key residues involved in enzyme catalytic activity. Arg4Ala and Gln295Ala mutations at the dimer interface reduced the catalytic activity | Ye et al.,(7) | X-ray crystallography, size-exclusion chromatography, and ultra-centrifugation |
| 10 | 128, 137, 185, 192 | In MERS CoV ^Mpro^, Lys137 and Glu290 form hydrophobic contacts with 185, and 128 forms van der Waals interactions with His8-Lys155. These residues are involved in control of the dimer interface. Gln192 forms hydrogen bond with a substrate (compound 6) at the active site | Tomar et al.,(8) | X-ray crystallography |
| 11 | 286 | In SARS-CoV M^pro^, Ser284-Thr285-Ile286/Ala mutations enhanced the catalytic machinery by enzyme dynamics | Lim et al.,(9) | X-ray crystallography |
| 12 | 27, 119 | Leu27 and Asn119 are involved in the formation of substrate binding site of SARS-CoV M^pro^ | Xue et al.,(10) | X-ray crystallography |
| 13 | 4 | In SARS-CoV M^pro^, salt bridge between Arg4-Glu290 stabilizes the dimer | Shi et al.,(11) | X-ray crystallography |
| 14 | 126 | In SARS-CoV M^pro^, Tyr126 is essential for dimer stability and for substrate catalytic machinery via aromatic and hydrophobic interactions between Tyr126 and Met6 and an aromatic interaction between Tyr126 with Phe140 | Shi et al.,(11) | X-ray crystallography |
| 15 | 126, 141 | In SARS-CoV M^pro^, 141-140-139 and 126 are directly involved in the dimer formation and regulation, which are required for catalysis | Shi et al.,(11) | X-ray crystallography, mutagenesis, and ultra-centrifugation |
| 16 | 141, 185 | In IBV M^pro^, Gly141 and Asp185 form a substrate binding pocket for the inhibitor N3 | Xue et al.,(10) | X-ray crystallography |
| 17 | 141, 192, 2, 4 | Leu141 and Gln192 contribute to the active site of SARS-CoV M^pro^ interact with authentic N-terminal residues (Gly2, Arg4), enhancing the activity | Xue et al.,(12) | X-ray crystallography |
| 18 | 4, 126 | In SARS-CoV M^pro^, Arg4Glu mutation produces weak dimer with no activity. Tyr126-M6 hydrophobic interaction stabilizes the dimer conformation | Wei et al.,(13) | Mutagenesis, enzyme assay, and analytical ultracentrifugation |
| 19 | 286, 295 | In SARS-CoV M^pro^, Ile286 with other critical dimerization residues form a channel to the catalytic center, which may play a role in regulating catalytic machinery.  The final C-terminal helix 293-306 is key for dimerization | Shi and Song,(14) | Mutagenesis, dynamic light scattering, CD and NMR spectroscopy |
| 20 | 4, 137 | In SARS-CoV M^pro^, Arg4 forms dimer interface with Lys137-Gln127 and Glu290 | Ghosh et al.,(15) | X-ray crystallography |
| 21 | 2, 4 | N-terminal (residues 1-4) truncation of SARS-CoV M^pro^ affects dimer and enzymatic activity | Hsu et al., (16) | Mutagenesis, enzyme assay, and analytical ultracentrifugation |
| 22 | 2, 4, 126, 286, 295 | Gly2, Arg4, Gly126, Glu286, and Gln295 are conserved and play key roles at the dimer interface in the M^pro^ structures of PEDV, TGEV, and HCoV-229E | Ye et al.,(7); Anand et al.,(17); Lee et al.,(18) | X-ray crystallography, size-exclusion chromatography, and ultra-centrifugation |
| 23 | 146 | In MERS-CoV M^pro^, Gly146 forms hydrogen bond with a substrate (compound 6) | Tomar et al., (8); Ionescu (19) | X-ray crystallography |
| 24 | 146, 150 | In SARS-CoV and MERS-CoV M^pro^s, conserved residues 143-147 (GSCGS motif) form oxyanion hole and important to initiate catalysis. F150 is a key for the structural stability of the catalytic center. | Wang et al., (20) | X-ray, mutational studies and enzyme  kinetics study |

SARS-CoV: Severe acute respiratory syndrome coronavirus, HKU4: Tylonycteris bat coronavirus HKU4, PEDV: Porcine epidemic diarrhea virus, HCOV-229E: Human coronavirus 229E, MERS: Middle East respiratory syndrome-related coronavirus, TGEV: Transmissible gastroenteritis virus, IBV: Infectious bronchitis virus

1. Douangamath, A., Fearon, D., Gehrtz, P., Krojer, T., Lukacik, P., Owen, C. D., Resnick, E., Strain-Damerell, C., Aimon, A., Abranyi-Balogh, P., Brandao-Neto, J., Carbery, A., Davison, G., Dias, A., Downes, T. D., Dunnett, L., Fairhead, M., Firth, J. D., Jones, S. P., Keeley, A., Keseru, G. M., Klein, H. F., Martin, M. P., Noble, M. E. M., O'Brien, P., Powell, A., Reddi, R. N., Skyner, R., Snee, M., Waring, M. J., Wild, C., London, N., von Delft, F., and Walsh, M. A. (2020) Crystallographic and electrophilic fragment screening of the SARS-CoV-2 main protease. *Nature communications* **11**, 5047

2. Kneller, D. W., Phillips, G., Weiss, K. L., Pant, S., Zhang, Q., O'Neill, H. M., Coates, L., and Kovalevsky, A. (2020) Unusual zwitterionic catalytic site of SARS-CoV-2 main protease revealed by neutron crystallography. *The Journal of biological chemistry*

3. Rut, W., Groborz, K., Zhang, L., Sun, X., Zmudzinski, M., Pawlik, B., Wang, X., Jochmans, D., Neyts, J., Mlynarski, W., Hilgenfeld, R., and Drag, M. (2020) SARS-CoV-2 M(pro) inhibitors and activity-based probes for patient-sample imaging. *Nature chemical biology*

4. Jin, Z., Du, X., Xu, Y., Deng, Y., Liu, M., Zhao, Y., Zhang, B., Li, X., Zhang, L., Peng, C., Duan, Y., Yu, J., Wang, L., Yang, K., Liu, F., Jiang, R., Yang, X., You, T., Liu, X., Yang, X., Bai, F., Liu, H., Liu, X., Guddat, L. W., Xu, W., Xiao, G., Qin, C., Shi, Z., Jiang, H., Rao, Z., and Yang, H. (2020) Structure of M(pro) from SARS-CoV-2 and discovery of its inhibitors. *Nature* **582**, 289-293

5. Zhang, L., Lin, D., Sun, X., Curth, U., Drosten, C., Sauerhering, L., Becker, S., Rox, K., and Hilgenfeld, R. (2020) Crystal structure of SARS-CoV-2 main protease provides a basis for design of improved alpha-ketoamide inhibitors. *Science (New York, N.Y.)* **368**, 409-412

6. Rathnayake, A. D., Zheng, J., Kim, Y., Perera, K. D., Mackin, S., Meyerholz, D. K., Kashipathy, M. M., Battaile, K. P., Lovell, S., Perlman, S., Groutas, W. C., and Chang, K. O. (2020) 3C-like protease inhibitors block coronavirus replication in vitro and improve survival in MERS-CoV-infected mice. *Science translational medicine* **12**

7. Ye, G., Deng, F., Shen, Z., Luo, R., Zhao, L., Xiao, S., Fu, Z. F., and Peng, G. (2016) Structural basis for the dimerization and substrate recognition specificity of porcine epidemic diarrhea virus 3C-like protease. *Virology* **494**, 225-235

8. Tomar, S., Johnston, M. L., St John, S. E., Osswald, H. L., Nyalapatla, P. R., Paul, L. N., Ghosh, A. K., Denison, M. R., and Mesecar, A. D. (2015) Ligand-induced Dimerization of Middle East Respiratory Syndrome (MERS) Coronavirus nsp5 Protease (3CLpro): IMPLICATIONS FOR nsp5 REGULATION AND THE DEVELOPMENT OF ANTIVIRALS. *The Journal of biological chemistry* **290**, 19403-19422

9. Lim, L., Shi, J., Mu, Y., and Song, J. (2014) Dynamically-driven enhancement of the catalytic machinery of the SARS 3C-like protease by the S284-T285-I286/A mutations on the extra domain. *PloS one* **9**, e101941

10. Xue, X., Yu, H., Yang, H., Xue, F., Wu, Z., Shen, W., Li, J., Zhou, Z., Ding, Y., Zhao, Q., Zhang, X. C., Liao, M., Bartlam, M., and Rao, Z. (2008) Structures of two coronavirus main proteases: implications for substrate binding and antiviral drug design. *Journal of virology* **82**, 2515-2527

11. Shi, J., Sivaraman, J., and Song, J. (2008) Mechanism for controlling the dimer-monomer switch and coupling dimerization to catalysis of the severe acute respiratory syndrome coronavirus 3C-like protease. *Journal of virology* **82**, 4620-4629

12. Xue, X., Yang, H., Shen, W., Zhao, Q., Li, J., Yang, K., Chen, C., Jin, Y., Bartlam, M., and Rao, Z. (2007) Production of authentic SARS-CoV M(pro) with enhanced activity: application as a novel tag-cleavage endopeptidase for protein overproduction. *Journal of molecular biology* **366**, 965-975

13. Wei, P., Fan, K., Chen, H., Ma, L., Huang, C., Tan, L., Xi, D., Li, C., Liu, Y., Cao, A., and Lai, L. (2006) The N-terminal octapeptide acts as a dimerization inhibitor of SARS coronavirus 3C-like proteinase. *Biochemical and biophysical research communications* **339**, 865-872

14. Shi, J., and Song, J. (2006) The catalysis of the SARS 3C-like protease is under extensive regulation by its extra domain. *The FEBS journal* **273**, 1035-1045

15. Ghosh, A. K., Xi, K., Ratia, K., Santarsiero, B. D., Fu, W., Harcourt, B. H., Rota, P. A., Baker, S. C., Johnson, M. E., and Mesecar, A. D. (2005) Design and synthesis of peptidomimetic severe acute respiratory syndrome chymotrypsin-like protease inhibitors. *Journal of medicinal chemistry* **48**, 6767-6771

16. Hsu, W. C., Chang, H. C., Chou, C. Y., Tsai, P. J., Lin, P. I., and Chang, G. G. (2005) Critical assessment of important regions in the subunit association and catalytic action of the severe acute respiratory syndrome coronavirus main protease. *The Journal of biological chemistry* **280**, 22741-22748

17. Anand, K., Palm, G. J., Mesters, J. R., Siddell, S. G., Ziebuhr, J., and Hilgenfeld, R. (2002) Structure of coronavirus main proteinase reveals combination of a chymotrypsin fold with an extra alpha-helical domain. *The EMBO journal* **21**, 3213-3224

18. Lee, C. C., Kuo, C. J., Ko, T. P., Hsu, M. F., Tsui, Y. C., Chang, S. C., Yang, S., Chen, S. J., Chen, H. C., Hsu, M. C., Shih, S. R., Liang, P. H., and Wang, A. H. (2009) Structural basis of inhibition specificities of 3C and 3C-like proteases by zinc-coordinating and peptidomimetic compounds. *The Journal of biological chemistry* **284**, 7646-7655

19. Ionescu, M. I. (2020) An Overview of the Crystallized Structures of the SARS-CoV-2. *Protein J*

20. Wang, H., He, S., Deng, W., Zhang, Y., Li, G., Sun, J., Zhao, W., Guo, Y., Yin, Z., Li, D., and Shang, L. (2020) Comprehensive Insights into the Catalytic Mechanism of Middle East Respiratory Syndrome 3C-Like Protease and Severe Acute Respiratory Syndrome 3C-Like Protease. *ACS Catal* **10**, 5871-5890
